# Natural Killer Cell Activity Test Helps to Suspect Aggressive Natural Killer Cell Leukemia - Diagnostic Challenge

**DOI:** 10.7150/ijms.77583

**Published:** 2023-01-16

**Authors:** Eunkyoung You, Chan-Jeoung Park, Min Young Lee, Young-Uk Cho, Seongsoo Jang, Jung-Hee Lee, Je-Hwan Lee, Kyoo-Hyung Lee

**Affiliations:** 1 Department of Laboratory Medicine, Inje University College of Medicine, Busan Paik Hospital, Busan, Korea.; 2 Department of Laboratory Medicine, Asan Medical Center, University of Ulsan College of Medicine, Seoul, Korea.; 3 Department of Laboratory Medicine, Kyung Hee University School of Medicine and Kyung Hee University Hospital at Gangdong, Seoul, Korea.; 4 Department of Hematology, Asan Medical Center, University of Ulsan College of Medicine, Seoul, Korea.

**Keywords:** Aggressive natural killer cell leukemia, Epstein-Barr virus, Hemophagocytic lymphohistiocytosis, NK cell activity

## Abstract

Aggressive natural killer cell leukemia (ANKL) is a rare disease with an aggressive clinical course. We aimed to assess the clinicopathological characteristics of the difficult to diagnose ANKL. During ten years, nine patients with ANKL were diagnosed. All the patients exhibited aggressive clinical course and underwent the BM study to rule out lymphoma and hemophagocytic lymphohistiocytosis (HLH). BM examination showed varying degrees of infiltration of neoplastic cells, which were mainly positive for CD2, CD56, cytoplasmic CD3 and EBV in situ hybridization. Five BM aspirates showed histiocytic proliferation with active heomphagocytosis. Normal or increased NK cell activity test results were obtained from 3 patients who were available for testing. Four had multiple BM studies until diagnosis. An aggressive clinical course and positive EBV in situ hybridization, often with associated secondary HLH, should raise the suspicion of an ANKL. Conducting additional supplementary tests such as NK cell activity and NK cell proportion would be helpful for the diagnosis of ANKL.

## Introduction

Aggressive natural killer cell leukemia (ANKL) is a systemic neoplastic proliferation of NK cells frequently associated with Epstein-Barr virus (EBV) and an aggressive course 1. ANKL is a rare disease that comprises of <1% of all non-Hodgkin lymphoma 1-3. ANKL is more prevalent among Eastern Asian populations than other ethnic populations, and develops mainly in the relatively young 4-6. ANKL has an extremely aggressive clinical course and shows poor prognosis, with less than 2 months of median survival 6, 7. Early and accurate diagnosis of ANKL is very important because of rapid progression.

The diagnosis of ANKL relies mainly on the integration of clinical features, laboratory findings, the morphologic and immunophenotypic features of neoplastic cells, and demonstration of the clonality of neoplastic NK cells 1,5-9. Clinically, patients with ANKL present with fever, constitutional symptoms, hepatosplenomegaly, and multiorgan dysfuntions. Cytopenia is common and hemophagocytic syndrome and coagulopathy are often presented. EBV is mostly detected in the neoplastic tissues, and is supportive for the diagnosis of ANKL. Detection of neoplastic NK cells in bone marrow (BM) and peripheral blood (PB) is required for the diagnosis of an ANKL. However, the low number of neoplastic cells at the initial presentation makes it extremely difficult for the correct diagnosis. We aimed to assess the clinicopathological characteristics of ANKL difficult to be diagnosed, by analyzing patients with ANKL in a single center.

## Materials and Methods

A total of 9 patients who were diagnosed with ANKL and treated at Asan Medical Center between January 2008 and March 2018 were included in this retrospective study. The retrieved data included the patients' age, sex, clinical signs, laboratory findings, histopathological findings of the BM, immunophenotypes of the BM and/or PB, EBV *in situ* hybridization, and results of the NK cell activity test and cytogenetic test,and outcomes. Detection of EBV-encoded RNA (EBER) was performed by *in situ* hybridization. To characterize the immunophenotype of the ANKL cells, a panel of antibodies for flow cytometry was used to detect various antigens including the leukocyte common antigen (CD45), NK-associated antigens (CD56, CD2, CD7, CD8, and CD16), T lymphocyte-associated antigens (surface CD3, cytoplasmic CD3, CD4, and CD5) and B lymphocyte-associated antigens (CD10, CD19, and CD22). Flow cytometry analyses were performed using the BD FAC SCanto™ II flow cytometer and BD CellQuest™ Pro Analysis software (BD Biosciences, San Jose, CA, USA). The NK cell activity test using K562 cells was performed as previously described 9. We used peripheral blood mononuclear cells (PBMCs) as effector cells and Fluorescein isothiocyanate-labeled K562 cells as target cells. NK-cell activity was calculated using the following equation: NK-cell activity (%) = (test lysis - spontaneous lysis) × 100/(maximum lysis - spontaneous lysis).

## Results

**Figure 1:**
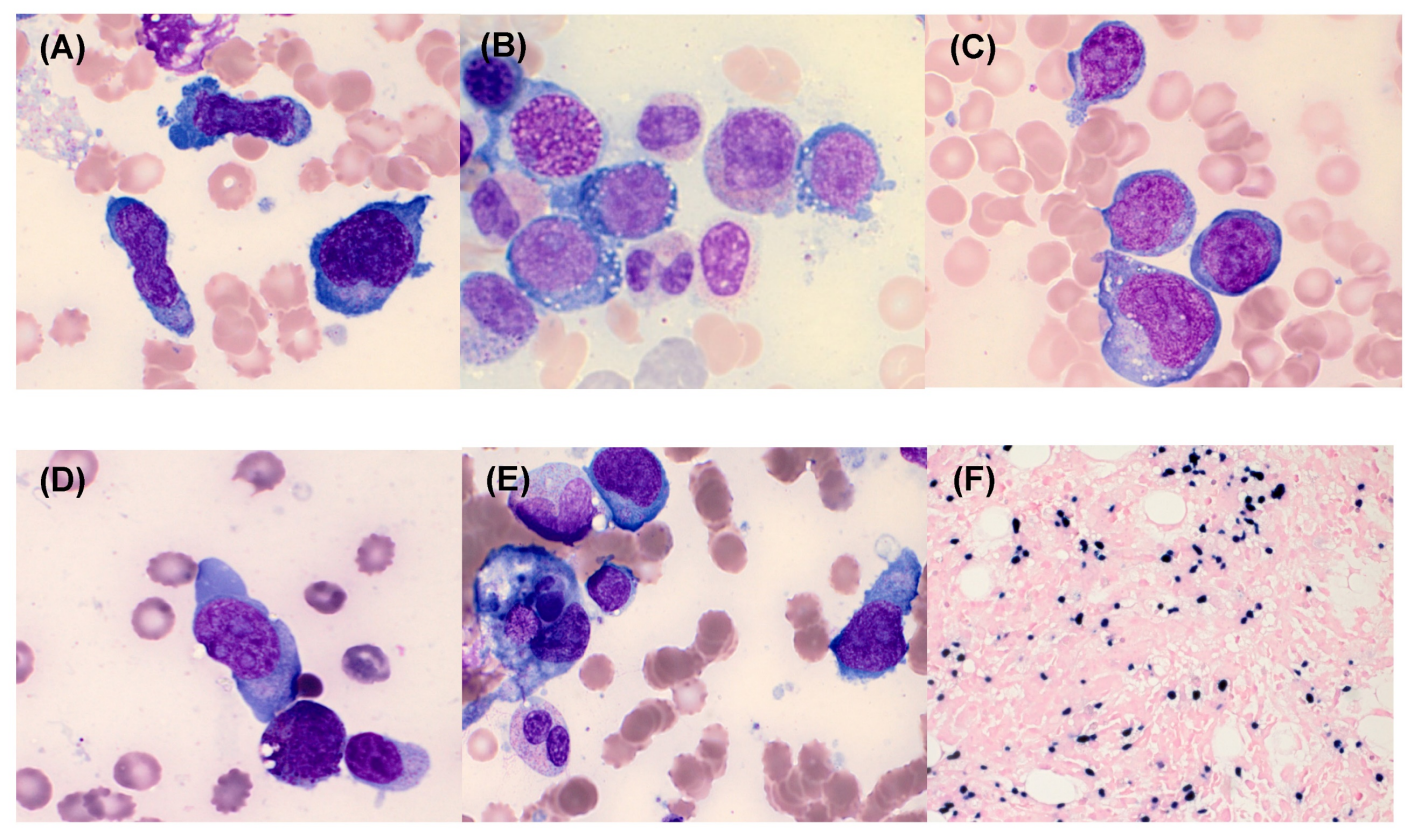
The bone marrow (BM) findings of aggressive natural killer cell leukemia. The neoplastic cells showed a medium-to-large size, a pleomorphic appearance **(A)**, moderate-to-abundant amount of basophilic cytoplasm, intracytoplasmic vacuoles** (B)**, few purple-red cytoplasmic granules in some neoplastic cells** (C)**, and abnormal nuclei with 1 or 2 nucleoli **(D)**. Hemophagocytosis was detected in BM aspirates** (E)** (Wright-Giemsa stain, ×1000). The neoplastic cells were EBER-positive on BM biopsy section (EBV *in situ* hybridization, ×400) **(F)**.

The clinical features and laboratory characteristics of the 9 patients with newly diagnosed ANKL are summarized in Table 1. Of the 9 patients, 8 were male the median age was 48 years (range: 23-73). All patients had clinical manifestations accompanied by fever and cytopenia. Most of the patients had anemia (8/9, 89%) and thrombocytopenia (9/9, 100%), while only 3 (33%) patients had leukopenia. All patients underwent BM study to rule out lymphoma (N=5) and haemophagocytic lymphohistiocytosis (HLH) (N=4). Hepatosplenomegaly was detected in 6 (67%) patients. BM examination showed varying degrees of infiltration of neoplastic cells (median, 35.6%; range: 18.4%-70.4%) with varying sizes from small to large (Figure 1). Neoplastic cells were positive for CD56 in all patients (9/9, 100%). They were positive for cytoplasmic CD3 in 4 cases (44%), CD2 in 7 cases (78%), CD7 in 6 cases (67%), CD16 in 4 cases (44%), and all negative for surface CD3, CD4, CD10, CD19, CD20, and CD22. EBER *in situ* hybridization was positive in 8 (89%) patients (Figure 1F). BM aspirates in 5 (56%) patients showed histiocytic proliferation with active hemophagocytosis. Complex cytogenetic abnormalities were identified in 6 (67%) patients. Among 3 patients wh o underwent NK cell activity test with PB, 1 patient showed normal NK cell activity and the other 2 patients showed increased NK cell activity. Lymphocyte subset test results showed markedly increased NK cell proportions in the PB of patients with ANKL, with an average of 72.2% (range: 55.2%-84.5%).

The median survival of the patients was 38 days (range: 1-120). Seven (78%) patients expired and the remaining 2 (22%) patients were lost in follow-up because they were transferred to other hospitals. Patients received a variety of chemotherapy regimens including CHOP-like (containing anthracycline and vincristine) and SMILE (dexamethasone, methotrexate, ifosfamide, L-asparaginase, and etoposide), as determined by the clinician.

**Figure 2:**
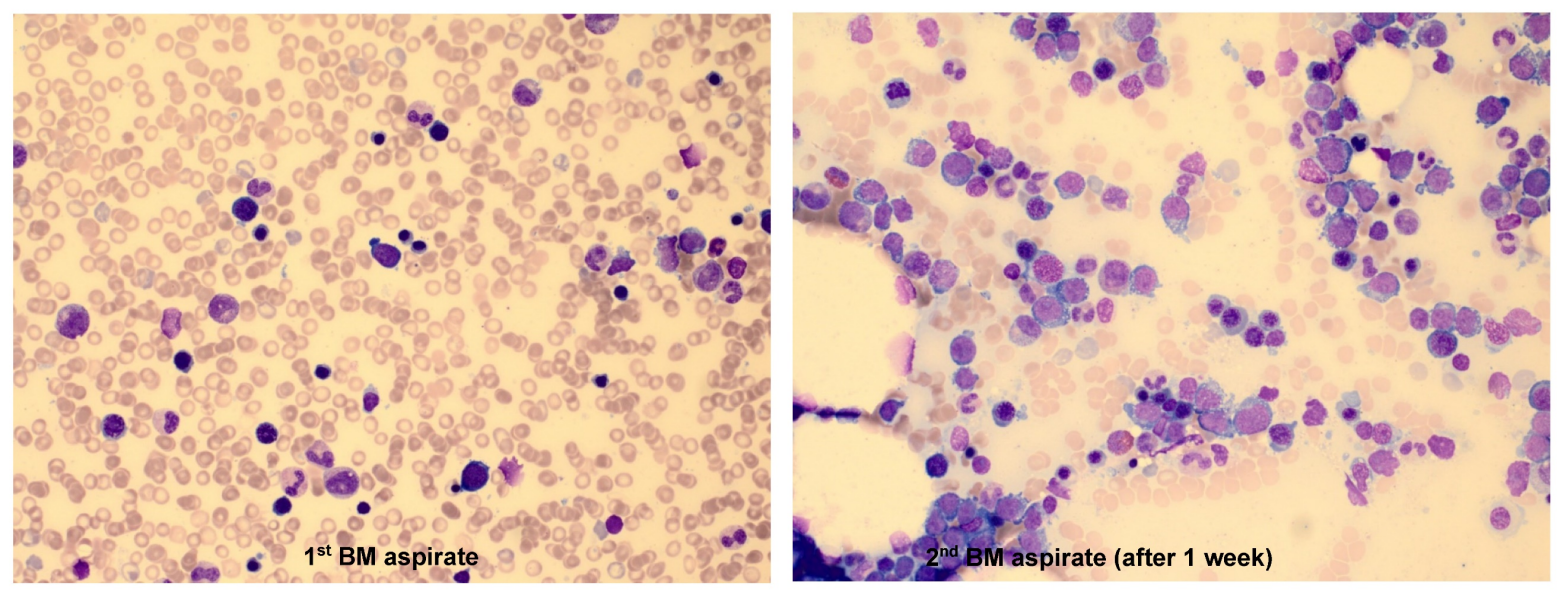
Bone marrow (BM) results from a chronic HBV carrier 23-year-old male who was admitted to the Department of Hematology with a history of fever and pancytopenia for few days. In the 1^st^ BM examination, the aspirate was partially hemodiluted and no definite neoplastic lymphoid cells were identified (1^st^ BM aspirate, Wright-Giemsa stain, ×400). The second BM examination was performed after 1 week due to the persistence of fever and pancytopenia, which showed that the aspirate was infiltrated with medium-to-large, primitive-looking lymphoid cells with prominent multiple nucleoli and abundant basophilic cytoplasm that comprised 18.4% among the BM nucleated cells. Some neoplastic cells had intracytoplasmic vacuoles (2^nd^ BM aspirate, Wright-Giemsa stain, ×400).

The characteristic findings were as follows: 1) Among 9 patients, 4 had multiple BM studies (≥ 2 times) until diagnosis, which was largely due to the insufficient amount of neoplastic cells in the first BM study. Obvious neoplastic cells were identified in the follow-up BM aspirates. One patient who did not show neoplastic cells in the first BM aspirates, had a markedly increased proportion of neoplastic cells (18.4%) in the second BM aspirates after 1 week (Figure 2). 2) Some patients presented with symptoms and signs of HLH such as unexplained fever, cytopenias, and hepatosplenomegaly. In the 'NK cell activity' test performed to diffretiate HLH in 3 patients, none of them showed a decrease in NK cell activity.

## Discussion

ANKL is an uncommon disease that occurs with higher frequency in Asians than in other ethnic groups 4-6, which is likely to be due to both genetic and environmental factors. ANKL is almost always associated with EBV, occurs almost exclusively in younger adults (median age, 39 years) and is slightly more common in males. Accordingly, our study population was also young and had a median age of disease onset of 48 (range: 23-73) and the proportion of male patients was 89%. In our study, the proportion of male patients is higher than that of female patients. However, this result should be interpreted carefully because the number of patients enrolled in this study is small. Age has been proposed as a prognostic factor 10, but it has not been validated 11. The primary indication for BM study was to rule out lymphoma in 5 patients and HLH in 4 patients, and most of the patients experienced fever, cytopenia, and hepatosplenomegaly. Therefore, when interpreting the BM study of patients with impression of lymphoma or HLH and an aggressive clinical course, the possibility of ANKL should be considered despite its rarity.

It is difficult to distinguish the neoplastic cells of ANKL observed in the BM aspirates from other lymphoid malignant cells solely based on the morphology, because atypical or activated lymphocytes are frequently observed in HLH. Another challenge in the diagnosis of ANKL is the low number of neoplastic cells in the initial BM study 8. Because ANKL progresses rapidly, numerous neoplastic cells can be observed in follow-up BM studies performed at 1-2 weeks after the initial BM study. In our study, 4 out of 9 patients had undergone multiple BM studies of 2 or more times before the diagnosis of ANKL. In one patient (case 1), there was no definite neoplastic cell infiltration observed in the first BM study, but in the second BM study performed 1 week later, 18.4% of neoplastic cells that were medium to large in size with prominent, multiple nucleoli and abundant basophilic cytoplasm were observed (Figure 2). ANKL is very rare, difficult to be diagnosed, and shows aggressive clinical course. Hence, when ANKL is suspected, it is important that BM study is performed again as early as possible.

Although flow cytometry offers many benefits in determining the characteristics of lymphoid neoplastic cells, its use is limited when the number of malignant cells is insufficient 8. In our patients, NK cell lineage disease could be suspected because all cells were positive for CD56, and most of them were also positive for CD2, CD7, CD16, and cytoplasmic CD3. Notably, the NK cell activity test was a helpful flow cytometry test in diagnosing ANKL. Three patients were tested for NK cell activity test using PB to rule out HLH, and its results were normal or increased NK cell activity. One of the diagnostic criteria for HLH is a decreased NK cell activity; however, all 3 patients did not exhibit reduced NK cell activity, and 2 of 3 patients showed increased NK cell activity. Furthermore, the lymphocyte subset test performed in parallel to NK cell activity test revealed that the proportions of NK cells in the above 3 patients were significantly increased (55.2%, 70.7%, and 84.5%). Thus, we knew that the NK cell activity did not decrease, but was either normal or increased, and that the proportion of NK cells in PB was markedly increased in ANKL. With these 2 findings, we could diagnose NK cell related lymphoid malignancies rather than the possibility of primary HLH. Thus peripheral NK cell proportions and NK cell activity test markedly contributed to the differential diagnosis of ANKL from primary HLH. For patients with aggressive clinical course and suspected HLH or lymphoma, normal or increased NK cell activity and increased peripheral NK cell proportion, will be helpful to diagnose ANKL.

The median survival time from ANKL diagnosis was approximately 38 days (range: 1-120 days), and except for 2 patients who were lost to follow-up, all patients expired. Because ANKL progresses rapidly and demonstrates poor prognosis, a fast and accurate diagnosis is most important. An aggressive clinical course and a strongly positive EBV *in situ* hybridization result, often with associated HLH, should raise the suspicion of ANKL. The immunophenotyping of neoplastic cells is essential, and additionally the NK cell activity test and peripheral NK cell proportion will also be helpful in diagnosing ANKL. For accurate diagnosis, an additional BM study might be necessary, if the patient is suspected to have ANKL based on clinical features, but no neoplastic cells are observed in the first BM study.

## Figures and Tables

**Table 1:** Clinical and laboratory characteristics of 9 patients with aggressive NK cell leukemia at diagnosis

N	Sex/age	r/o	H-S	WBC-Hb-PLT	Neoplastic cells	Cellularity	Immunophenotype	EBV	2^nd^ HLH	NK activity	NK cell	Outcome	Multiple BM study	Karyotype
				×10^9^/L, g/L, ×10^9^/L	BM (%)	%					PB (%)	Days	number	
1	M/23	LM	+	2.8-94-36	18.4%	70%	CD2, cCD3, CD56	+	+	NL	55.20%	HD 98*	+/3	46,XY,i(8)(q10),inc[4]/72<3n>,XXY,+1,-2,+3,dic(3;14)(p25;q32),+4,+5, i(8)(q10),+10,dic(11;19)(q23;p13.1),+15,-16,+19,+19,+mar,inc[1]/46,XY[45]
2	M/34	HLH	+	0.9-80-12	22.8%	10%	CD2, CD7, CD8, CD56	+	+	I	70.70%	E (HD 16)	-	46,Y,t(X;4)(q22;q25)[11]/46,XY[9]
3	M/32	HLH	+	9.3-94-47	35.2%	60%	CD2, CD7, CD16, CD56	+	+	I	84.50%	HD 120*	+/2	46,X,-Y,t(8;9)(q13;p13),add(20)(q13.3),+mar[9]/46,XY[11]
4	M/44	HLH	+	7.6-82-9	70.4%	90%	CD2, CD7, CD56, CD16	-	-		NT	E (HD 22)	+/2	46,XY,del(17)(p13)[4]/46,XY[21]
5	F/72	LM	-	3.7-85-92	52.6%	30%	CD2, cCD3, CD7, CD56	+	-		NT	E (HD 50)	-	46,XX,del(9)(p22),add(12)(p13),der(20)t(1;20)(q21;q13.3)^4^/46,XX[20]
6	M/59	LM	+	4.3-102-21	34.8%	60%	cCD3, CD7, CD16, CD56	+	+		NT	E (HD 16)	-	46,XY[20]
7	M/67	HLH	+	5.2-61-123	26.4%	40%	CD2, CD7, CD56	+	+		NT	E (HD 1)	+/4	47,-X,-Y,add(3)(q27),+9,del(13)(q12q22),+del(13)(q12q22),add(18)(q12),+mar[20]
8	M/73	LM	-	5.8-164-99	20.8%	60%	cCD3, CD56	+	-		NT	E (HD11)	-	47,XY,add(4)(q35),del(11)(q23),-18,add(22)(p11.2),+2mar[6]/46,XY[14]
9	M/67	LM	-	6.3-107-82	39.0%	75%	CD2, CD16, CD56	+	-		NT	E (HD5)	-	46,XY,del(6)(q21),del(7)(q22q32),del(13)(q12q14),add(15)(q22),add(19)(p13.3)[7]/ 46,sl,-Y,der(3)t(3;12),-del(7)(q22q32),add(7)(p15)x2,del(11)(q13q21),-12,+2mar[2]/92,sdl1x2[2]/46,sl,-Y,i(7)(q10),+mar[1]/46,XY[8]

*Lost in follow-up. Abbreviations: N, number; r/o, rule out; H-S, hepatospleomegaly; WBC, white blood cell; Hb, hemoglobin; PLT, platelet; BM, bone marrow; EBV, Epstein-Barr virus; HLH, hemophagocytic lymphohistiocytosis; NK, natural killer cell; PB, peripheral blood; M, male; F, female; LM, lymphoid malignancy; CD, cluster of differentiation; c, cytoplasmic; NL, normal; I, increased; NT, not tested; HD, hospital day; E, expired.
